# Supplementary material for: The Efficacy of Hybrid Vaginal Ovules for Co-Delivery of Curcumin and Miconazole against Candida albicans
Source: Pharmaceutics. 2024 Feb 23;16(3):312. doi: 10.3390/pharmaceutics16030312 (PMC10974232; doi:10.3390/pharmaceutics16030312)
Supplement: Supplementary file 1 [file pharmaceutics-16-00312-s001.zip › pharmaceutics-2844546-supplementary.pdf]

Supplement Materials: Efficacy of hybrid vaginal ovules for co-delivery of curcumin and miconazole against *Candida albicans*

Brenda Maria Silva Bezerra, Sara Efigênia Dantas de Mendonça y Araújo, José de Oliveira Alves-Júnior, Bolívar Ponciano Goulart de Lima Damasceno, João Augusto Oshiro-Junior

Table S1. Thermogravimetric parameters of U-PEO, CUR, MCZ, CUR/MCZ 1:1, and U-PEO ovules loaded with the drugs.

Group	Step 1		Step 2		Step 3	
	Tonset–Tendset (ºC)	Weight loss (%)	Tonset–Tendset (ºC)	Weight loss (%)	Tonset–Tendset (ºC)	Weight loss (%)
U-PEO	50.20–200.70	7.53	268.33–431.39	56.24	446.76–545.79	12.78
CUR	239.12–433.28	56.01	440.73–900.00	17.71	–	–
MCZ	180.42–214.16	20.49	219.42–356.58	65.72	361.40–900.00	10.04
CUR/MCZ 1:1	214.40–370.75	47.58	375.00–900.00	29.27	–	–
U-PEO/CUR	45.08–272.08	5.67	273.00–436.85	62.71	446.05–900	10.25
U-PEO/MCZ	40.10–259.70	5.45	261.46–429.78	62.36	435.05–900.00	13.33
U-PEO/CUR/MCZ	43.50–266.65	6.44	267.54–502.98	69.65	505.60–900.00	4.35

Table S2. DTA parameters of CUR, MCZ, CUR/MCZ 1:1, and hybrid precursor PEO combined with CUR and MCZ.

Group	Peak 1		Peak 2	
	Tpeak (ºC)	ΔH (J/g)	Tpeak (ºC)	ΔH (J/g)
CUR	186.41	-77.11	381.34	23.79
MCZ	185.29	-42.81	208.25	252.56
CUR/MCZ 1:1	161.79	-58.99	–	–
PEO/CUR 1:1	111.67	-4.66	348.84	14.17
PEO/MCZ 1:1	–	–	194.52	97.19

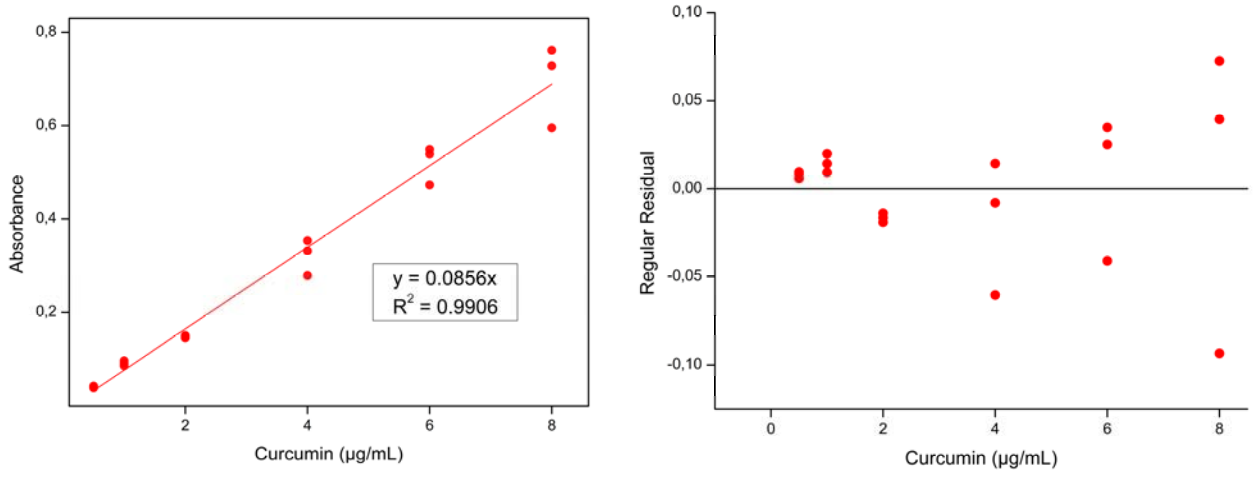

(**a**) (**b**)

Figure S1. Linear regression (**a**) and residual plot (**b**) of curcumin in acetate buffer pH 4.2 added with ethanol and Tween80.

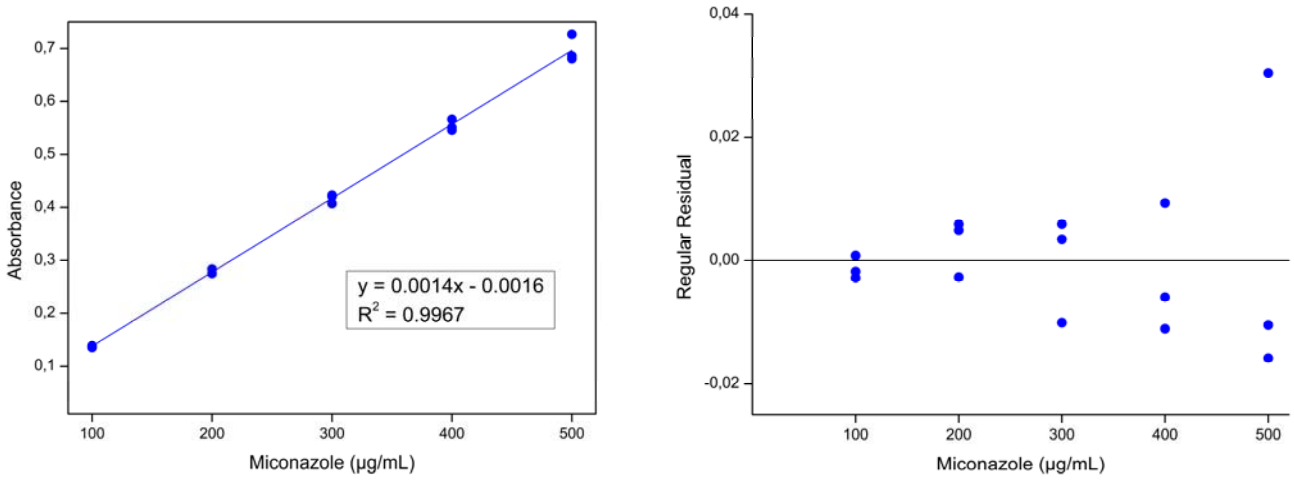

(**a**) (**b**)

Figure S2. Linear regression (**a**) and residual plot (**b**) of miconazole in acetate buffer pH 4.2 added with sodium lauryl sulfate.

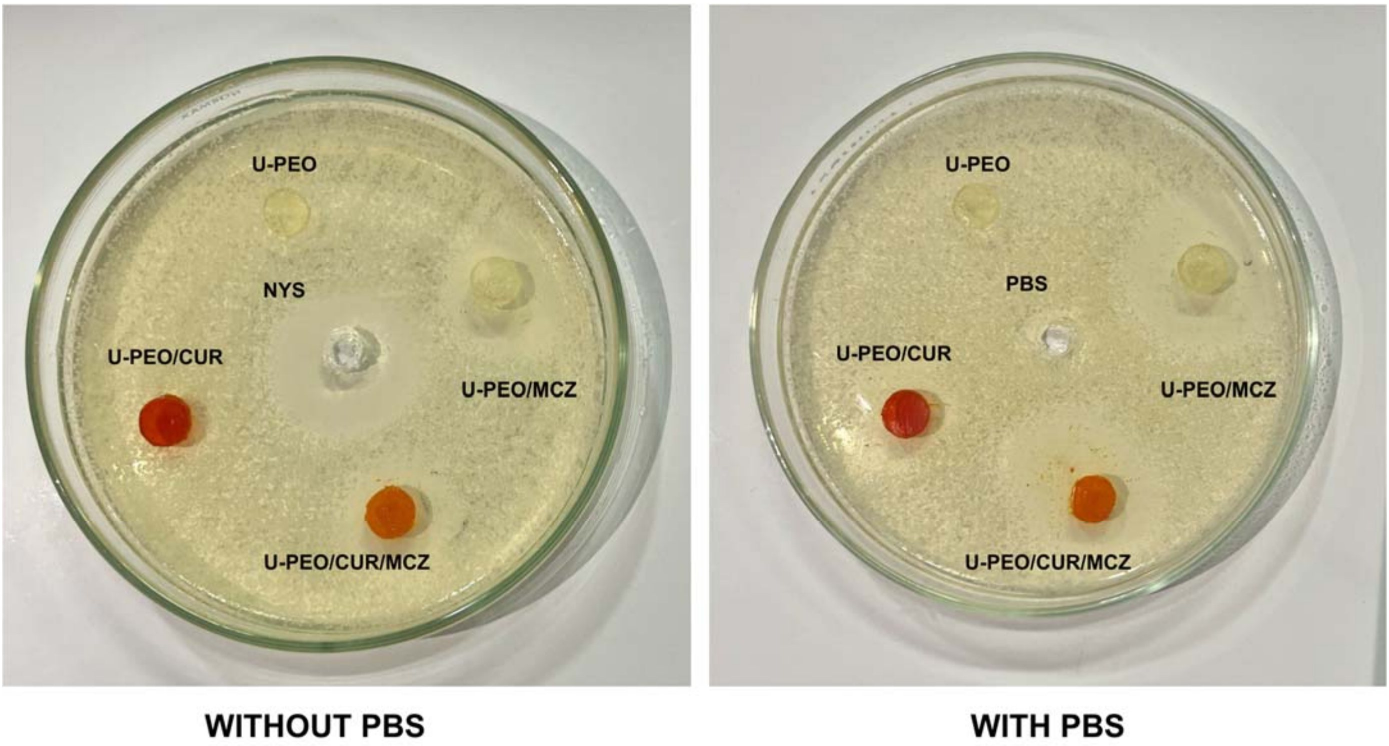

(**a**) (**b**)

Figure S3. Agar diffusion assay of U-PEO, U-PEO/CUR, U-PEO/MCZ, and U-PEO/CUR/MCZ ovules without placing PBS (**a**) and placing 100 μL of PBS (**b**) on the sectioned materials. NYS (nystatin) and PBS (Phosphate buffer) as control groups.
